# Advances in imaging ultrastructure yield new insights into presynaptic biology

**DOI:** 10.3389/fncel.2015.00196

**Published:** 2015-05-22

**Authors:** Joseph J. Bruckner, Hong Zhan, Kate M. O’Connor-Giles

**Affiliations:** ^1^Cell and Molecular Biology Training Program, University of Wisconsin-MadisonMadison, WI, USA; ^2^Laboratory of Cell and Molecular Biology, University of Wisconsin-MadisonMadison, WI, USA; ^3^Laboratory of Genetics, University of Wisconsin-MadisonMadison, WI, USA

**Keywords:** presynapse, active zone, synaptic vesicle, electron microscopy, ultrastructure

## Abstract

Synapses are the fundamental functional units of neural circuits, and their dysregulation has been implicated in diverse neurological disorders. At presynaptic terminals, neurotransmitter-filled synaptic vesicles are released in response to calcium influx through voltage-gated calcium channels activated by the arrival of an action potential. Decades of electrophysiological, biochemical, and genetic studies have contributed to a growing understanding of presynaptic biology. Imaging studies are yielding new insights into how synapses are organized to carry out their critical functions. The development of techniques for rapid immobilization and preservation of neuronal tissues for electron microscopy (EM) has led to a new renaissance in ultrastructural imaging that is rapidly advancing our understanding of synapse structure and function.

## Introduction

A functional nervous system requires the establishment of neural circuits that reliably execute complex tasks and adapt to stimuli that change over time. Neural circuit function, in turn, depends on the proper formation, function, and plasticity of synaptic connections between component neurons. Defects in the organization and function of synapses underlie diverse neurological disorders including autism, depression, and memory loss. Although synapses were first hypothesized at the turn of the twentieth century, their study required significant advances in microscopy and they were not directly observed until the development of the electron microscope in the 1950s. The minute size and intricate molecular composition of synapses has made them an experimental challenge since their discovery. Increasingly, this challenge is being met through the application of innovative techniques for imaging at ultrastructural and near-ultrastructural levels.

The presynaptic terminal enables neuronal communication by releasing neurotransmitter in response to Ca^2+^ influx through voltage-gated calcium channels activated by the arrival of an action potential. Neurotransmitter released from the presynaptic terminal into the narrow synaptic cleft converts electrical input into a chemical output received by a postsynaptic cell through binding of neurotransmitter to postsynaptic receptors. Proper neurotransmitter release requires accurate functional organization at the presynaptic terminal such that neurotransmitter-containing synaptic vesicles (SVs) are clustered within the terminal, docked at release sites, and rapidly fused with the synaptic membrane within a microdomain of increased Ca^2+^ concentration. Neurotransmitter release properties must be both reliable and plastic to maintain the scalability of synaptic strength in response to varying inputs. Although the postsynaptic specialization plays an equally important role in neuronal communication, it is well reviewed elsewhere (in Feng and Zhang, 2009; MacGillavry et al., 2011; Gold, 2012; Iasevoli et al., 2012). In this review, we focus on the contribution of ultrastructural imaging techniques to a mechanistic understanding of the signal-sending side of the synapse, the presynaptic terminal.

**Figure 1:**
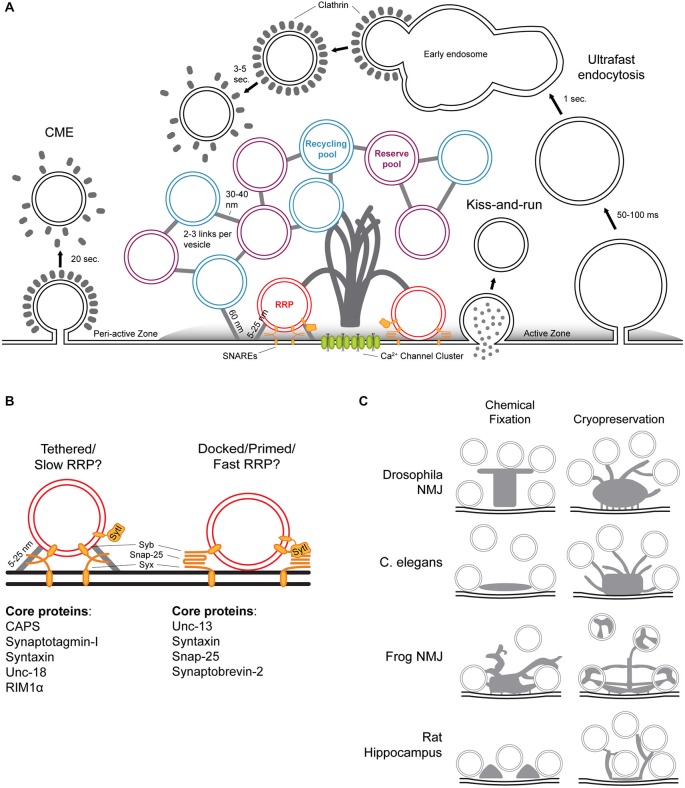
**Structure-function relationships of the presynaptic terminal. (A)** Diverse presynaptic terminals have a number of common structural characteristics visible in electron micrographs. The active zone (AZ) membrane is delineated by its electron-dense lipid bilayer. Complex cytoskeletal filaments project from the AZ membrane into the presynaptic cytoplasm and are often visible as an electron-dense projection. SVs are 40–60 nm in diameter and organized into three functionally defined pools: the reserve pool (purple), recycling pool (blue), and readily releasable pool (RRP; red). SVs of the reserve and recycling pools are typically linked to one another by 2–3 thin proteinaceous tethers 30–40 nm in length, and occasionally linked to the AZ membrane by longer filaments of roughly 60 nm in length. The reserve and recycling pools are morphologically intermixed and therefore defined primarily by their mobility in functional assays. RRP vesicles are tethered or docked at the membrane in close proximity to clusters of voltage-gated calcium channels at the base of the dense projection. Three modes of endocytosis are hypothesized for recovery of SVs following exocytosis: clathrin-mediated endocytosis (CME), kiss-and-run, and ultrafast endocytosis. The newly described ultrafast endocytosis involves the formation of 80-nm diameter vesicular intermediates within 50–100 ms after stimulus that fuse with early endosomal compartments within 1 s after stimulus. SVs are then reformed from the early endosome in a clathrin-dependent manner 3–5 s post stimulus. **(B)** The RRP includes SVs tethered to the AZ membrane by short filaments 5–25 nm in length and SVs in direct contact with the membrane. Although the exact molecular composition of SV tethers is unknown, some of the AZ proteins responsible for regulating SV tethering and docking/priming are known. **(C)** Cryopreservation of synapses reveals the morphological intricacies of dense projection structure previously masked by chemical fixation and dehydration. Although the unique functional requirements of distinct synapses within and between species likely underlie observed differences in morphology, most Dense projections (DPs) comprise a central core and radiating filaments of varying lengths that contact of distinct functional pools.

Early electron microscopy (EM) studies first identified many of the now well-known features of synapses, including SVs and post-synaptic densities (De Robertis and Bennett, 1955; Palay and Palade, 1955). Although synaptic ultrastructure varies across species and neuronal subtype, the general functions described above are shared among all synapses and generate commonalities in synaptic architecture (Zhai and Bellen, 2004). Neurotransmitter release is accomplished through a complex network of molecules at the presynaptic active zone (AZ; Couteaux and Pécot-Dechavassine, 1970; Südhof, 2012). The AZ provides both a structural and molecular foundation for synaptic activity, and is identifiable in all synapses as a strip of electron-dense presynaptic plasma membrane tightly apposed to a postsynaptic electron density comprising neurotransmitter receptors and associated cytoskeletal proteins. The presynaptic terminal also contains large clusters of SVs sorted into distinct pools, commonly subdivided into the following: (1) the reserve pool that maintains release during prolonged activity, (2) the recycling pool; and (3) the readily-releasable pool (RRP), which can be further subdivided into SVs “tethered” to the AZ membrane by short filaments and “docked” SVs in direct contact with the AZ membrane absent any visible tethers. The RRP is defined as SVs that are released upon mechanical stimulation with hypertonic sucrose and thought to represent the population accessed upon Ca^2+^ influx during normal physiological activity (Figures 1A,B; Rosenmund and Stevens, 1996).

Although conventional EM techniques employing chemical fixatives have been successfully applied for decades and yielded tremendous insights into synaptic structure and cell biology, recent work has demonstrated that in hydrated samples, preservation of fragile cellular ultrastructure is difficult to achieve with chemical fixatives due to slow penetration and diffusion throughout the cell. This results in significant alterations to neuronal morphology and ultrastructure, including changes in the distribution of functional components, especially, as discussed below, synaptic vesicles and the filamentous cytoskeleton. Rapid freeze preservation has been applied for decades in many organisms and tissues, beginning with “freeze-fracture” and “freeze-etching” experiments that split the phospholipid bilayer to reveal membrane faces (Moor et al., 1961; Steere, 1989; reviewed in Heuser, 2011). Recent high-pressure freeze/freeze substitution (HPF/FS) techniques have improved ultrastructural preservation in dissected or cultured neuronal tissues, as well as intact organisms including Caenorhabditis elegans and *Drosophila* larvae (Dubochet et al., 1988; Landis et al., 1988; Rostaing et al., 2004; Fouquet et al., 2009; Stigloher et al., 2011; McDonald et al., 2012). Through the application of high pressures (2100 bar) at the freezing point within milliseconds, HPF/FS maintains the benefit of suspending the biological sample in a near-native state while allowing vitrification to penetrate up to 200 microns into tissue. Following this rapid immobilization, chemical fixatives are slowly substituted into the tissue as it is warmed to room temperature over several days. Alternatively specimens can be maintained and imaged at −170°C for “cryo-EM,” eliminating the need for chemical preservation following immobilization, although these techniques are typically limited to cultured cells and thin isolated tissues (Dubochet, 1995; Zuber et al., 2005). Beyond HPF/FS, recent EM work has advocated a more “proteocentric” approach to immobilizing and staining synaptic tissues. By avoiding the OsO_4_ traditionally used to preserve lipids at the expense of protein, it has become possible to visualize the molecular conformation of the proteinaceous AZ and cytomatrix (Burette et al., 2012). Despite demonstrated effects on synaptic ultrastructure, conventional EM preparations utilizing aldehyde fixatives still remain an important complementary approach to rapid cryofixation. Although the speed of cryofixation may assist the immobilization of transient events, HPF/FS is certainly not gentle—it is possible that the application of high pressure and transition to vitrification, no matter how fast, may alter the endogenous distribution of synaptic components (Südhof, 2012). Therefore any conclusions drawn from the morphology of the synaptic ultrastructure must take into consideration existing light-level imaging, electrophysiological, genetic, and biochemical studies. These recent advances in EM have combined with super-resolution light microscopy to address synaptic function by characterizing the constitutive components of synapses *in situ*. Here we discuss our expanded understanding of presynaptic structure and function with a focus on those discoveries made possible by advances in ultrastructural imaging.

## A Complex Network of Proteinaceous Filaments Organizes Presynaptic Terminals

A primary function of the presynaptic terminal is to cluster SVs into functional pools that can release neurotransmitter upon the arrival of action potentials, sustain release, and modulate it in response to changing stimuli. In fact, recent reconstructions of the molecular organization of synaptic terminals suggest that a significant portion of the presynaptic proteome is devoted to trafficking SVs (Wilhelm et al., 2014). Furthermore, SVs themselves are intricate proteinaceous structures to which a large body of work has been devoted (Takamori et al., 2006). Studies in hippocampal slice preparations, cultured neurons, and dissociated synaptosomes have suggested that a network of filaments linking SVs is the primary structural determinant of SV clustering and organization. Rapid freezing/freeze etching enabled the first observation of this complex network of filaments radiating from both synaptic vesicles and the presynaptic membrane (Landis et al., 1988). Changes observed in the network of SV tethers at the presynaptic terminal in response to electrical or mechanical stimulus suggest that they are highly dynamic, supporting a role in mediating vesicle release dynamics. Recent observations in C. elegans suggest the filamentous cytomatrix not only tethers SVs but also provides a general structural framework for organelles within the terminal (Stigloher et al., 2011; Astro and de Curtis, 2015).

HPF/FS EM and cryoelectron tomography have begun to yield a clearer picture of the filamentous cytomatrix linking SVs (Rostaing et al., 2006; Fernández-Busnadiego et al., 2010; Jiao et al., 2010; Stigloher et al., 2011; Helmprobst et al., 2015). Tomographic reconstruction of HPF/FS-prepared tissue slices provides a boost in axial resolution relative to conventional EM that further facilitates the visualization of fine synaptic filaments. In contrast to higher estimates from single thin sections, it appears that each SV (40–60 nm in diameter) is linked to one or two neighboring vesicles by short filaments (30–40 nm in length), and that longer filaments (50–60 nm in length) connect the SV network to the AZ membrane (Figure 1A). These filament lengths are consistent in different organisms and neuronal subtypes, including the electric ray electric organ, frog and zebrafish NMJs, rat cerebrocortical synaptosomes, and mouse cerebellar cortex or hippocampal slices (Landis et al., 1988; Hirokawa et al., 1989; Rostaing et al., 2006; Siksou et al., 2007; Fernández-Busnadiego et al., 2010; Helmprobst et al., 2015).

The filamentous cytomatrix appears to increase in complexity with proximity to the AZ membrane, an observation that is easily reconciled with the need for more complex vesicle trafficking and release machinery near SV release sites. The complex cytomatrix of the active zone (CAZ) is highly adapted to the functional requirements of specific synapses, and in many cases can be observed as a projection from the AZ membrane in electron micrographs (Figures 1A,C). For example, ribbon synapses in the vertebrate retina and cochlea are so named for the presence of large ribbon-like, electron-dense projections that extend into the presynaptic cytoplasm where they tether large pools of SVs. These specialized structures are thought to enable neurotransmitter release rates sufficient for retinal neurons to encode stimulus intensity in the strength of release rather than release rate, and in many neurons DPs (DPs) appear to be responsible for clustering synaptic vesicles near the AZ membrane where they can be rapidly trafficked for release (Lenzi and von Gersdorff, 2001; Parsons and Sterling, 2003; tom Dieck and Brandstätter, 2006; Weimer et al., 2006; Zampighi et al., 2008). The complexity of DPs varies dramatically and can range from a simple linear amorphous density within several nm of the cell membrane to extensive filamentous projections extending several hundred nm into the interior of the presynaptic bouton (Zhai and Bellen, 2004). It is likely that neurotransmitter release in general is regulated by common mechanisms, but that differences in the morphology of DPs represent the diversification of a central structure/function theme to diverse niches.

The morphology of the CAZ is closely linked to its functional capabilities and impaired synaptic function has long been associated with structural defects. To that end, a significant investment has been made in characterizing presynaptic DPs in diverse synaptic subtypes. The application of HPF/FS to neuronal tissues has advanced an entirely new perspective on the morphology of DPs (Figure 1C). For example, at the AZ of the *Drosophila* neuromuscular junction (NMJ), DPs were initially termed “T-bars” due to their shape in electron micrographs, a thick pedestal topped by a platform that clustered synaptic vesicles. With HPF/FS, the *Drosophila* NMJ T-bar is in fact filamentous—an observation easily reconciled with light-level studies demonstrating that the T-bar component and CAST/ELKS/ERC homolog Bruchpilot adopts an elongate conformation (Fouquet et al., 2009). In C. elegans, conventional EM studies suggested that the DP was a plaque-like structure that extended across the width of the AZ but remained shallow within the cytoplasm (Zhai and Bellen, 2004). In fact, as in *Drosophila*, HPF/FS preservation reveals that the C. elegans DP is composed of elongated filaments extending up to 100 nm into the cytoplasm of the synaptic terminal (Kittelmann et al., 2013). Vertebrate DP structures are highly variable depending on synaptic subtype, ranging from the ribbons described above to minute electron densities regularly spaced between docked SVs in mammalian central synapses (Gray, 1961; Pfenninger et al., 1972; Dick et al., 2003). In rat hippocampal slice cultures prepared using HPF/FS, DPs lose their regularity and take on a sparse, filamentous appearance (Siksou et al., 2007; Zhao et al., 2012b). In a recent electron tomographic analysis of zebrafish NMJ AZs, DPs appear as a complex arbor of filaments radiating from a dense central core, a structure that is more reminiscent of DPs at the frog or *Drosophila* NMJ than vertebrate central synapses (Helmprobst et al., 2015). Although previous work has struggled to reconcile diversity in DP structure with an apparent common functional purpose, in coming years the application of HPF/FS techniques to each of these systems will likely elucidate the shared and divergent roles of DPs in synaptic function (Zhai and Bellen, 2004).

## High-Resolution Microscopy is Clarifying the Molecular Organization of the Presynaptic Cytomatrix

As clear images emerge of the filamentous nature of DPs, ultrastructural and near-ultrastructural studies have begun to address the molecular correlates of these structures. Quick-freeze deep-etch EM expands on the freeze fracture preparations described above through vacuum sublimation of up to 10 μm of ice from the freeze-fractured membrane surface, enabling enhanced preservation of the topology of 3D structures (Heuser, 2011). Deep-etch EM of SVs incubated with Synapsin *in vitro* revealed fine structures linking SVs and, combined with single-molecule reconstruction, suggested that the molecular conformation of Synapsin matched that of the SV linkers (Hirokawa et al., 1989). Although this correlation with the distribution and conformation of the SV protein Synapsin I led to the hypothesis that the synaptic web was composed of this protein, triple knock-out of all Synapsin isoforms does not appear to disrupt the filamentous nature of the cytomatrix, either between vesicles or between vesicles and the AZ (Siksou et al., 2007). Therefore, although Synapsin may be a critical component of SV tethers, other molecules must be involved in their formation and maintenance (Takei et al., 1995; Siksou et al., 2007). On the other hand, incubating the synapse in tetanus toxin significantly reduces the number of filaments near the AZ membrane, suggesting that the formation of this subset of the cytomatrix may be regulated by the synaptic vesicle SNARE protein Synaptobrevin, and that distinct regions of the filamentous cytomatrix are separable in their molecular regulation (Fernández-Busnadiego et al., 2010).

**Figure 2:**
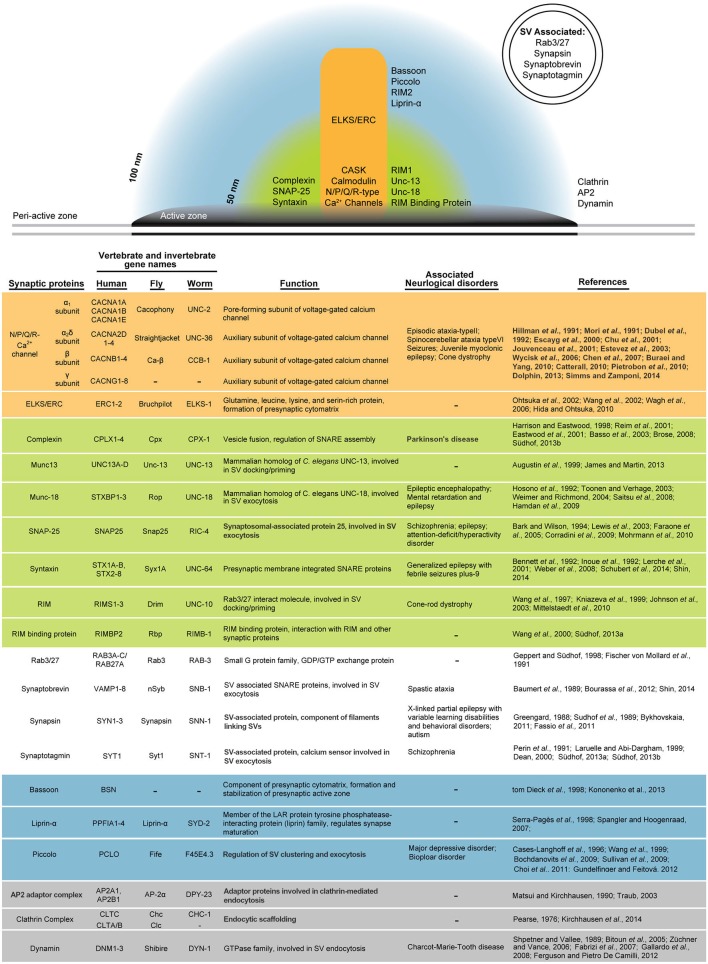
**A highly conserved network of proteins organizes presynaptic function**. Presynaptic proteins can be grouped into zones within the AZ based on studies of their location. Although the structural complexity of the presynapse is not illustrated (see Figure 1), it can be generally divided into the dense projection (orange), an AZ-proximal zone extending to approximately 50 nm from the membrane (green), an AZ-distal zone extending to approximately 100 nm from the membrane (blue), the peri-active zone beyond the electron dense AZ membrane (gray), and SVs that may transiently occupy any of the other zones. Presynaptic proteins discussed in this review are grouped according to their predominant localization and described in detail. For each molecule, conservation in multiple species is indicated with the gene name from humans, flies, and worms. Links between presynaptic proteins and human neurological disorders are summarized.

The precise organization and exact function of DPs remain enigmatic. However, a highly conserved network of interacting proteins comprising the CAZ has been well defined in many model organisms, including Piccolo/Aczonin, Bassoon, Rab3 interacting molecules (RIMs), Unc-13, RIM binding protein (RBP), liprin-α, and CAST/ELKS/ERC (Figure 2; Dresbach et al., 2001; Rosenmund et al., 2003; Zhai and Bellen, 2004; Jin and Garner, 2008; Bruckner et al., 2012). A large body of work has furthered our understanding of how each component contributes functionally to the behavior of the synapse and is reviewed elsewhere (Haucke et al., 2011; Gundelfinger and Fejtova, 2012). However, a detailed understanding of CAZ morphology has been hindered by the detection limits of conventional light microscopy where the diffraction limit of light restricts lateral resolution to between 200 and 300 nm and axial resolution to 500 nm (Abbe, 1873; Pawley, 2010; Bianchini et al., 2015). To bypass the diffraction limit and achieve nanometer resolution, both immuno-EM and super-resolution microscopy have been applied to the question of protein localization within the presynaptic CAZ. Stochastic optical reconstruction microscopy (STORM) and the related technique photo-activated light microscopy (PALM) take advantage of “on” and “off” states of photo-switchable fluorophores to temporally distribute localization information and computationally reconstruct the center of each diffraction spot (Betzig et al., 2006; Rust et al., 2006). STORM was used to establish the axial distribution of synaptic proteins within the presynaptic terminal in mouse olfactory bulb and cortex synapses, where RIM1 was found between 20 and 50 nm from the presynaptic membrane and the related Piccolo and Bassoon proteins were detected between 20 and 100 nm from the membrane (Dani et al., 2010). Combining immuno-gold labeling and HPF/FS EM has enabled localization of synaptic proteins with nanometer resolution in an ultrastructural context (Rostaing et al., 2006; Weimer et al., 2006). Such a study in cerebellar synapses found that RIM1 and Munc-13 localize closest to the plasma membrane at approximately 20 nm, while Piccolo adopts an L-shaped conformation at the tip of the DP where it extends to cover the distal AZ between 40 and 80 nm from the synaptic membrane (Limbach et al., 2011). In *Drosophila*, immuno-EM detecting two distinct epitopes of Bruchpilot demonstrated that it adopts an elongated conformation at the DP core (Fouquet et al., 2009). These data inform compelling hypotheses about the role of these proteins in synaptic function, where Piccolo and Bassoon regulate SV trafficking at the AZ perimeter; Munc13–1, RIM1, and CAST1 have a direct role in priming in support of SV release; and CAST/ELKS/ERC/Bruchpilot bridges these two compartments.

CAZ proteins are also responsible for clustering Ca^2+^ channels in close proximity to SV release sites. Although decades of work has generated a better understanding of Ca^2+^ dynamics at the presynaptic AZ (Llinás et al., 1992; DiGregorio et al., 1999; Frank et al., 2009), the precise localization of Ca^2+^ channel subunits at sub-AZ resolution has only recently been determined using the application of advanced imaging techniques, and remains a significant challenge. Immunogold labeling of CA3 pyramidal cells prepared using rapid freeze/freeze fracture demonstrated that CaV_2.1_ channels are distributed nonrandomly, clustering at presumptive SV release sites (Holderith et al., 2012). A similar technique applied to rat cerebellar parallel fibers also observed small clusters of CaV_2.1_ channels between 50 and 100 nm in diameter (Indriati et al., 2013). Super-resolution imaging approaches are also beginning to reveal the molecular determinants of Ca^2+^ channel localization. Stimulated emission depletion (STED) microscopy boosts resolution by using an additional depletion laser to suppress fluorescence emission around the focal point, and has been applied to *in vivo* imaging of neuronal tissues where it can localize synaptic proteins with 60 nm lateral resolution (Hell and Wichmann, 1994; Nägerl et al., 2008; Liu et al., 2011; Urban et al., 2011). STED microscopy was instrumental in detecting a disorganization of Ca^2+^ channel punctae in inner ear hair cells lacking Bassoon, demonstrating its role in organizing the precise localization of Ca^2+^ channels at AZs (Frank et al., 2010).

Detection efficiency in immuno-EM is limited by many factors, including embedding material, epitope preservation, lack of specific antibodies and difficulty achieving adequate tissue penetration. Correlative light and EM microscopy (CLEM) has begun to address these issues by combining the advantages of EM with those of fluorescent protein microscopy. In one approach, this is accomplished using a hydrophilic resin and cryo-EM to preserve protein fluorescence post-embedding. A laser integrated in the EM column allows registration of fluorescent signal from ultrathin sections in electron micrographs (Agronskaia et al., 2008; Faas et al., 2013). Watanabe et al. developed nano-fEM, which employs STED and PALM super-resolution imaging on ultrathin sections that are subsequently imaged in a scanning electron microscope. This method was first employed to study the localization of presynaptic Liprin-α/SYD-2 in C. elegans where it was found to localize specifically to the DP in agreement with previous results using immuno-EM (Yeh et al., 2005; Watanabe et al., 2011). In many cases the development of antibodies against synaptic proteins has proven difficult, limiting the ability to localize these molecules with nanometer resolution. To overcome this obstacle, many studies have employed the overexpression of fluorescently tagged proteins to determine precise localization. However, the degree to which overexpressed proteins represent endogenous localization and intermolecular interactions is often unclear. Recent developments in genome engineering are enabling many groups to circumvent the caveats of overexpression by facilitating efficient endogenous tagging of proteins (Harrison et al., 2014). The generation of targeted double-strand breaks with CRISPR-Cas9 allows for highly efficient homology directed repair that can incorporate exogenous sequences, including fluorescent or small epitope tags, into any gene of interest, and promises to advance our understanding of the molecular structure of synapses.

Most CAZ proteins are expressed as multiple isoforms, effectively multiplying the complexity of the molecular machinery in a way that most studies have yet to address. *Drosophila* Bruchpilot is expressed as two distinct isoforms, Brp-190 and Brp-170. Two-color STED imaging at the NMJ with 50-nm resolution determined that Bruchpilot isoforms are arranged in an alternating array around Ca^2+^ channel clusters, where together they provide a structural link between the RRP at the base of the DP and tethered vesicles at the AZ periphery (Matkovic et al., 2013). The application of STORM to this same system has further revealed that each AZ comprises roughly 15 heptameric units interspersed with “free” unclustered protein (Ehmann et al., 2014). Bruchpilot clusters are encircled by *Drosophila* RIM-Binding Protein, which promotes Ca^2+^ channel clustering and Ca^2+^-dependent neurotransmitter release. STED microscopy was used to localize two distinct Rim-Binding Protein epitopes with 50 nm resolution, revealing an elongated conformation with its C-terminus positioned near Ca^2+^ channel clusters and its N-terminus closer to the membrane-distal C-terminus of Bruchpilot (Liu et al., 2011).

The boost in resolution gained from super-resolution microscopy has also unmasked phenotypes that suggest specific functional roles for other molecules in the regulation of CAZ morphology. In a *Drosophila* model of amyotrophic lateral sclerosis (ALS), flies overexpressing the DNA/RNA-binding protein Fused in Sarcoma exhibit a subtle malformation of AZ Bruchpilot clusters that would have been impossible to detect with traditional confocal microscopy (Lanson et al., 2011; Shahidullah et al., 2013). Similar defects were detected in mutants lacking the *Drosophila* PDZ domain-containing protein Dyschronic using STED microscopy, suggesting a role in CAZ assembly (Jepson et al., 2014). Another super-resolution technique, structured illumination microscopy (SIM), illuminates samples with patterned light to create moiré fringes for the computational reconstruction of features below the resolution limit, and improves lateral resolution by a factor of two (Gustafsson, 2000). SIM microscopy has been employed to clarify the role of microtubules and their associated proteins in linking the CAZ to the cytoskeleton, revealing the association of microtubules with nearly all AZ Bruchpilot clusters (Lepicard et al., 2014).

## Synaptic Vesicles are Organized into Functional Pools with Morphological Correlates

Models of SV trafficking postulate three pools of vesicles distinguished primarily by their mobility rather than their physical localization within the terminal: the reserve pool, the recycling pool and the RRP (Rizzoli and Betz, 2005; Denker and Rizzoli, 2010). Although these pools were originally defined electrophysiologically, the application of high-resolution electron tomography to preparations that preserve native structure has enabled a detailed morphological definition of synaptic vesicle pools that is closely related to their functional properties.

In the bouton interior up to several hundred nanometers from the AZ membrane, clusters of SVs linked by thin filaments are organized in many distinct groupings of varying size—up to 20 distinct clusters in some systems (Fernández-Busnadiego et al., 2010). Although SVs in these clusters appear morphologically similar they can be distinguished by their mobility into two populations: the recycling pool which is continuously replenished and trafficked to the AZ membrane and the reserve pool which is only accessed under extreme conditions (Haucke et al., 2011). The RRP is operationally defined as the population of vesicles released in response to mechanical stimulus with a hypertonic sucrose solution. In rat cerebrocortical synaptosomes, quantification of the morphological difference after hypertonic stimulation only observed release of vesicles tethered to the AZ membrane by more than two tethers under 5 nm in length. Except for two cases in which SVs were observed linked to the AZ membrane by a narrow fusion pore, SVs making direct contact with the AZ membrane were not observed in this preparation (Fernández-Busnadiego et al., 2010). Combined with studies in other cell types and organisms that detect morphological docking more frequently, this work suggests that SVs tethered to the AZ membrane by short 5–25 nm filaments comprise the RRP, and that direct “docking” contact between an RRP vesicle and the AZ membrane may represent a transient event that proceeds rapidly to neurotransmitter release. It is also possible that vesicles of the RRP oscillate stochastically between primed and un-primed states. If this is the case, synaptic probability of release may be a key determinant in the distribution of the RRP found in tethered vs. docked states. In support of this idea, conical electron tomography, which uses more complex tilt geometry to reduce missing volume in tomographic reconstructions, was used to demonstrate that in cortical synapses approximately 75% of vesicles in contact with the presynaptic membrane are hemi-fused (Lanzavecchia et al., 2005; Zampighi et al., 2005). In this hemi-fused state, the inner leaflet of the SV membrane remains intact but the outer leaflet is continuous with the inner leaflet of the AZ membrane. Contact between SVs never involved hemi-fusion, supporting the model of proteinaceous tethers between vesicles (Zampighi et al., 2006). Further, recent electrophysiological work at the murine calyx of Held suggests that the RRP is composed of two vesicle populations with distinct release kinetics (Figure 1B). The “fast” RRP is released within 1 ms of stimulation and the “slow” RRP is released approximately 4 ms after stimulation (Chen et al., 2015). It is possible that vesicles in the “fast” RRP are docked, while vesicles in the “slow” RRP are tethered. The future application of rapid freeze imaging techniques to address this model will help determine the morphological identity of vesicles within the RRP, and define how each contributes to the release probability of individual synapses.

## Morphological Docking Precedes Synaptic Vesicle Fusion

Rapid release of neurotransmitter is thought to require physical docking and molecular priming to tightly synchronize membrane fusion with presynaptic Ca^2+^ influx, yet the morphological correlate of molecular priming has remained elusive. A critical step that must occur before coordinated fusion is the UNC-13-mediated assembly of the soluble N-ethylmaleimide-sensitive factor attachment protein receptor (SNARE) complex, where zippering of trans-SNAREs forces the fusion of SV and AZ membranes. SNARE assembly is thought to be the final molecular priming step enabling SV fusion competence, and decades of work have addressed the molecular and biophysical mechanisms that underlie SV fusion (reviewed in Südhof, 2013a,b; Kaeser and Regehr, 2014). However, key structure-function relationships remain unclear. In work using aldehyde fixatives, the lack of a vesicle docking phenotype in synapses lacking UNC-13, despite complete loss of both spontaneous and evoked fusion, led to a distinction between vesicle priming and docking, where priming must occur downstream of docking but before exocytosis (Aravamudan et al., 1999; Richmond et al., 1999; Varoqueaux et al., 2002). The application of HPF/FS EM tomography to these samples revealed a loss of SV docking in Munc13 KO synapses, raising the possiblity that vesicle contact with the presynaptic membrane (docking) may be the structural correlate of functional priming (Siksou et al., 2009). This remains an open question.

Cryo-electron tomography of HPF/FS-immobilized rat synaptosomes are clarifying the structure-function relationships underlying SV docking/priming. RIM1α regulates the formation of short SV-AZ membrane tethers, and through these structures regulates the size of the RRP (Fernández-Busnadiego et al., 2013). Synapses lacking CAPS, Synaptotagmin I, Syntaxin I, and UNC-18 also lack tethered SVs and exhibit impaired neurotransmitter release, suggesting that these molecules may regulate tethering upstream of docking/priming (Figure 1B; Gracheva et al., 2010; Imig et al., 2014).

The proximity of the RRP to Ca^2+^ channels is a key determinant of release probability and kinetics, and the coupling of the RRP to Ca^2+^ channel clusters is a critical aspect of SV docking/priming. Although the sub-AZ localization of Ca^2+^ channel clusters relative to other molecules has not been directly determined with nm resolution, the combination of genetic and biochemical studies with STED super-resolution light microscopy suggests that they likely reside at the base of the DP, directly opposite of neurotransmitter receptor clusters (Liu et al., 2011; Graf et al., 2012; Matkovic et al., 2013). Computational simulations of Ca^2+^ influx and SV release suggest that SVs more distal to Ca^2+^ channel clusters may represent the slow RRP detected electrophysiologically (von Gersdorff and Matthews, 1994; Heidelberger et al., 1994; Jiao et al., 2010; Chen et al., 2015). At the fly NMJ, the majority of SVs tethered to the AZ membrane are linked to the AZ membrane by 5–25 nm filaments within 50 nm of the DP base (Jiao et al., 2010). During depolarization, vesicles at this distance from a Ca^2+^ channel cluster are likely exposed to Ca^2+^ concentrations between 50 and 100 μM. Therefore, if the necessary molecular priming steps have been achieved, tethered SVs should be rapidly released as part of the RRP during evoked release in physiological conditions.

The molecular nature of SV docking/priming appears to vary somewhat between synapses, likely due to the distinct release requirements of different neurons. For example, although a number of synaptic scaffolding proteins are conserved, it appears that many of the Ca^2+^ sensing and SV priming functions carried out in other cells by Synaptotagmin, RIMs, and Munc13 may be carried out by the protein Otoferlin in cochlear inner hair cells (IHCs). Recent electron tomography of IHC synapses demonstrated that loss of Otoferlin in IHCs results in a reduction of short SV-AZ membrane tethers, the same structures thought to be the structural correlate for the RRP in the rat cortex (Gracheva et al., 2010; Imig et al., 2014). The ability of large IHC synapses to sustain a RRP replenishment rate of around 700 SVs per second at each AZ, compared to 70 SVs per second per AZ in rat bipolar cells likely necessitates specialized machinery (Singer and Diamond, 2006; Pangršic et al., 2012). Although the exact molecular mechanism by which Otoferlin facilitates such high rates of neurotransmitter release remains unclear, it is possible that its many C2 domains enable sophisticated Ca^2+^ sensing and more rapid membrane bending than in other cell types (Pangršic et al., 2012).

Extensive electron tomography of the frog NMJ has enabled McMahan and colleagues to link the structural characteristics of the presynaptic terminal to SV docking, where the DP was classified into three distinct structural layers and the stepwise handoff of SVs between the macromolecular complexes that comprise each layer is thought to achieve SV docking (Figure 1C). The first class of AZ macromolecules occupies roughly 15 nm closest to the AZ and contains structures termed pegs, ribs, and beams; these structures likely contain Ca^2+^ channels and the molecules that regulate their distribution relative to vesicle docking sites. Beams extend lengthwise down the elongate AZ structure where they are linked to docked SVs by filamentous ribs that make contact with the presynaptic plasma membrane via short pegs (Harlow et al., 2001). The next 15 nm from the AZ is the intermediate layer, which contains steps and spars spaced at regular intervals and may mediate the first transition to morphological docking before contact with the proximal class of AZ material. Steps extend centrally from the AZ-proximal beams, and are linked to docked SVs by filamentous spars. The final class of macromolecules includes masts, which extend 30 nm from steps as a coiled bundle of 4–9 strands, booms, and topmasts. Booms appear to link the inner structure of the DP to the distal surface of docked vesicles, whereas filamentous topmasts extend furthest from the central mast to contact clustered, undocked SVs, possibly facilitating the replacement of recently fused vesicles with this clustered population (Szule et al., 2012). Furthermore, electron tomographic examination of the luminal structure of docked SVs revealed that SVs contain a consistently oriented internal skeleton that enables appropriate orientation of a docking vesicle, possibly through interactions with sparsely distributed vesicular proteins required for SV fusion (Harlow et al., 2013). Elements of this ultrastructural arrangement appear to be conserved across phyla, although again the presence of certain structural features may be dependent on the functional requirements of the synaptic subtype. At the fly NMJ, electron tomography of synapses prepared by HPF/FS revealed that DPs are composed of a central core, extensions, and legs, an organizational scheme that is conserved in other insects including locusts (Jiao et al., 2010; Leitinger et al., 2012). Relative to the frog NMJ, the central core may be analogous to the mast, step, and beam; the extensions similar to the spar, boom, and topmast; and the legs the equivalent of the ribs and pegs tightly associated with Ca^2+^ channels at the base of the DP. The combination of these high-resolution structural analyses with protein localization studies will further elucidate the contribution of each macromolecular structure to SV trafficking and neurotransmitter release.

## Advanced Ultrastructural Imaging Techniques Enable the Preservation of Exocytic and Endocytic Events

In the 1980s, Heuser and Reese combined a carefully timed electrical stimulus with rapid freezing of neuronal tissues, enabling the first observation of exocytic and endocytic intermediates (Heuser and Reese, 1981). The improved spatial resolution of EM tomography and ability of HPF/FS to immobilize tissue on rapid time scales has enabled the study of transient events in the SV cycle, especially exocytosis and endocytosis. In many situations the direction of these transient events can be difficult to determine conclusively—whether an “omega” shape at the synaptic membrane represents vesicle fusion or endocytosis is not inherently obvious. These “snapshots” have frozen fusion events at each of the hypothesized intermediate steps—a slight invagination of the presynaptic membrane, SVs linked to the synaptic cleft by a narrow fusion pore, and fully spherical vesicles making membrane contact with the AZ membrane (Fernández-Busnadiego et al., 2010). “Omega” figures are observed in 20% of synapses within 15 ms of ontogenetic stimulus and only rarely in unstimulated controls. The appearance of many of these structures immediately following stimulus suggests that they represent SVs in the act of full collapse into the AZ membrane. Optogenetic stimulus on short time scales appears to only release vesicles making morphological contact with the AZ membrane (Fernández-Busnadiego et al., 2010; Watanabe et al., 2013). Stimulus strength and type likely has a significant effect on the proportion of the RRP that is released in these experiments as mechanical stimulus with sucrose solutions depletes SVs that are tethered to the membrane by short (5–25 nm) filaments in addition to those docked at the membrane.

Neurons exhibit extreme polarity—in some cells the presynaptic terminal may be located at a distance hundreds of times the length of the cell body. Therefore, sustained activity requires local recycling of molecules and organelles, especially SVs. Early experiments using their “freeze slammer” technique led Heuser and Reese to develop a model of SV endocytosis where SVs are retrieved by bulk endocytosis (Heuser and Reese, 1973). One extensively studied mode of SV endocytosis is termed clathrin-mediated endocytosis (CME). CME is slow (around 20 s after stimulation), occurs at the AZ periphery, and requires clathrin and a number of adapter proteins (Heuser and Reese, 1973, 1981; Watanabe et al., 2013; reviewed in McMahon and Boucrot, 2011). Following exocytosis, vesicular membrane proteins are trafficked to the AZ periphery where they can be recycled to maintain SV identity during endocytosis. In PC12 neuroendocrine cells, the post-fusion diffusion of the vesicular acetylcholine transporter (VAChT) was tracked using a combination of iPALM and freeze-fracture EM. Following exocytosis, VAChT localization extends several hundred nanometers from the fusion site, where it is trapped at preformed clathrin endocytic structures through an interaction between its cytoplasmic tail and the adaptor protein AP2 (Kim and Hersh, 2004; Sochacki et al., 2012). By imaging these minute nascent clathrin structures and associated VAChT with iPALM and EM, Sochacki and colleagues determined that preassembled clathrin domes are present at high density in order to prevent the diffusion of vesicular proteins far from release sites (Sochacki et al., 2012).

Subsequent experiments have led to a model in which neurotransmitter release can be accomplished by either full collapse into the synaptic membrane or partial “kiss-and-run” release (Alabi and Tsien, 2013). Kiss-and-run is hypothesized to be the release of neurotransmitter through a narrow fusion pore formed by the force of the AZ cytoskeleton restraining against SNARE-mediated full collapse, enabling more rapid recovery of synaptic vesicles. Whether a given SV undergoes kiss-and-run or full collapse is thought to be dictated by binding of Ca^2+^ to Synaptotagmin (Syt) C2 domains (Wang et al., 2003). Previous work has employed multiple methods to define kiss-and-run release in several different neuronal and non-neuronal cell types, including FM dye destaining, pHlourin-based pH responses, quantum dot dequenching, whole cell capacitance recordings, measurements of postsynaptic receptor currents, and amperometry (Ceccarelli et al., 1972; Richards et al., 2005; Zhang et al., 2009; reviewed in Alabi and Tsien, 2013). However, measurements of kiss-and-run obtained by these techniques have been interpreted in different, sometimes conflicting, ways and a consensus on the degree to which a kiss-and-run mechanism is accessed during normal activity in diverse synaptic subtypes has not emerged (Granseth et al., 2006; Wu et al., 2007).

Expanding on the freeze slammer technique, Erik Jorgensen, Christian Rosenmund, Shigeki Watanabe, and colleagues have combined optogenetic stimulation with HPF/FS tissue preservation to preserve the state of the synaptic terminal within milliseconds after stimulating vesicular release. This “flash and freeze” technique finally enables visualization of discrete, rapid events, bringing the temporal resolution of EM into a range approaching that of electrophysiological measurements. In cultured mouse hippocampal neurons, flash and freeze has enabled the definition of three distinct forms of endocytosis: ultrafast (50–100 ms), fast (1 s), and slow (20 s). Ultrafast endocytosis occurs at the AZ edge, is clathrin-independent, and requires Dynamin and actin for scission and trafficking of endocytosis products (Watanabe et al., 2013). Vesicular products generated by ultrafast endocytosis are recycled through endosomal pathways and reformed into synaptic vesicles in a clathrin-dependent manner within 5–6 s (Watanabe et al., 2014). Trafficking of reformed synaptic vesicles within the terminal also appears to depend on actin and the size and release probability of the synapse, where larger, higher-probability synapses require F-actin for fine positioning and translocation of SVs across larger distances and at faster speeds (Rust and Maritzen, 2015). Although ultrafast endocytosis appears distinct from kiss-and-run exocytosis, it likely represents an adaptation that enables fast firing rates incompatible with slow clathrin mediated endocytosis (Watanabe et al., 2013). Despite the large body of work supporting multiple models of endocytosis, the degree to which each is employed during normal neuronal function remains unclear. Recent work suggests that the terminal may be able to shift between slow and ultrafast endocytosis depending on the vesicular requirements as dictated by temperature or neuronal activity (Micheva and Smith, 2005; Granseth et al., 2006; Renden and von Gersdorff, 2007; Watanabe et al., 2014; Kononenko and Haucke, 2015). RRP SVs appear to utilize multiple endocytic modes following exocytosis. Fast microwave-assisted aldehyde fixation and photoconversion of endocytic vesicles labeled with FM1–43 dyes revealed that, after release, 50% of the RRP vesicles are retrieved close to the AZ within 4 s after stimulation in a clathrin-independent manner. Within 30 s, the rest of the RRP is endocytosed through large intermediate vesicles, and a small proportion of the pool rejoins the RRP (Schikorski, 2014).

As discussed above, following ultrafast endocytosis SVs are reformed from endosomal compartments in a clathrin-dependent manner. Is endosomal SV reformation similar to CME in other ways? SIM microscopy was used to image endogenously tagged Clathrin heavy chain (Chc) and clathrin adaptor subunit α-Adaptin (α-Ada). Dynamin photoinactivation blocks relocalization of Chc and α-Ada from the center of the bouton to the periphery after stimulus, suggesting that SV budding from synaptic endosomes is stabilized by Dynamin (Kasprowicz et al., 2014). Therefore, SV reformation from synaptic endosomes employs a molecular mechanism similar to CME, which may have contributed to the omission of ultrafast endocytosis from earlier interpretations and models.

## Synaptic Ultrastructure is Plastic

Presynaptic specializations are plastic in response to changes in network activity, and homeostatic changes in molecular composition and activity can alter neurotransmitter release on short time scales. The structural basis of synaptic plasticity has long been elusive, but is likely mediated in part by the dynamic nature of presynaptic architecture. For example, pharmacologically silencing neurons in hippocampal slice cultures with bath application of the Na^+^ channel blocker tetrodotoxin increases the size of the presynaptic bouton, AZ, postsynaptic density, and RRP (Murthy et al., 2001). At the *Drosophila* NMJ, irreversible blockage of glutamate receptors via application of the wasp venom philanthotoxin or loss of the glutamate receptor subunit GluRIIA leads to the homeostatic increase in the amount of neurotransmitter released to maintain normal levels of neuronal activity in the network. This increase is accompanied by morphological changes visible with super-resolution microscopy, in this case STED, including an increase in the size of the RRP and complexity of the CAZ (Weyhersmüller et al., 2011). Immuno-EM has also been used to detect activity-dependent rearrangement of CAZ proteins on short time scales. Following high K^+^-induced depolarization, SV proteins including SV2, Synaptophysin, Synapsin, and Synuclein redistribute towards the AZ, Piccolo and Bassoon remain stable, and RIM1 relocates away from the AZ membrane (Tao-Cheng, 2006). The structural malleability of the CAZ is achieved in part by degradation of proteinaceous synaptic tethers via the ubiquitin-proteasome system (UPS). Although the complete molecular composition of the filamentous cytomatrix remains unclear, the levels of synaptic proteins including RIM, Munc13, and Synapsin are modulated by the UPS in an activity-dependent manner (Waites et al., 2013). Other CAZ proteins likely provide the link between changes in neural activity and the ubiquitination of synaptic proteins, and Bassoon and Piccolo have emerged as likely candidates. Through the loss of negative regulation of the E3 ubiquitin ligase Siah1, reduction of Piccolo and Bassoon protein levels lead to the degradation of SV-associated proteins including Synaptophysin, VAMP2, SV2, and Synapsin IA (Waites et al., 2013).

Chemically induced long-term potentiation (LTP) is also associated with ultrastructural changes at the synapse (Wojtowicz et al., 1994). A model for the plasticity mechanisms associated with learning and memory, LTP involves both pre- and postsynaptic molecular changes. When LTP is induced over the course of 10 min by application of the potassium channel blocker tetraethylammonium (TEA), the occurrence of omega-shaped invaginations drastically increases, most likely because increased neurotransmitter release dramatically improves the odds of immobilizing a full collapse fusion event. LTP also appears to induce an elongation of the presynaptic membrane that is coordinated with increasing complexity of postsynaptic spines, changes that depend on the priming factor Munc13–1 (Zhao et al., 2012a). In addition to trafficking new molecular complexes from the cell body, neurons may also accomplish molecular plasticity through local protein translation, and STED imaging is now being employed to address this question (Zhang et al., 2014). Although there is now significant evidence supporting translation of synaptic transcripts in the cell soma and dendrites, evidence in support of translation in the presynaptic compartment after the synapse primarily comes from studies employing the local application of protein synthesis inhibitors (Hsiao et al., 2014; reviewed in Akins et al., 2009). Presynaptically translated transcripts remain difficult to identify, but include the neuropeptide sensorin in Aplysia, and β-catenin in dissociated rat hippocampal cultures (Liu et al., 2003; Lyles et al., 2006; Wang et al., 2009; Taylor et al., 2013). Although fluorescent *in situ* hybridization (FISH) detected messenger RNA (mRNA) of the synaptic proteins Synaptobrevin and Synaptotagmin only in the soma and occasional dendrite of cultured hippocampal neurons, applied to other synaptic proteins in diverse neuronal subtypes, the increased resolution gained from STED-FISH may reveal previously overlooked mRNA localization in presynaptic terminals (Zhang et al., 2014). Parallel ultrastructural studies employing cryopreservation and tomography may hold promise in addressing the failure to detect polyribosomes presynaptically.

## Conclusions

The accumulation of minute changes in large synaptic subpopulations lies at the root of many neurologic disorders (van Spronsen and Hoogenraad, 2010; Chen et al., 2014; Deák, 2014). The ability to rapidly preserve synaptic biology in a state as close to life as possible for ultrastructural analysis is rapidly expanding our understanding of synaptic structure-function relationships. Applying these approaches to disease models promises to shed new light on the structural and molecular underpinnings of synaptic disorders. Despite their shared functions, synapses are structurally diverse both between organisms and between neuronal subtypes within an organism. Therefore, a comprehensive understanding of neuronal function requires the application of high-resolution imaging techniques to diverse synapses. Advances in imaging including HPF/FS, cryo-electron tomography, and super-resolution imaging have enabled studies of synaptic biology to move increasingly to experiments *in vivo* and even in intact, behaving organisms. Now, just decades after the first direct visualization of synapses, the revolution in advanced imaging techniques is shedding new light on synapse biology.

## Conflict of Interest Statement

The authors declare that the research was conducted in the absence of any commercial or financial relationships that could be construed as a potential conflict of interest.
